# Comparing Three Profoundly Influential Prognostic Scores in Cirrhotic Patients with Acute-on-Chronic-Liver Failure Admitted to the ICU: Prediction of One-Month Mortality—A Retrospective Cohort Study

**DOI:** 10.3390/diagnostics13203160

**Published:** 2023-10-10

**Authors:** Shih-Hua Lin, Wei-Ting Chen, Ming-Hung Tsai, Wei-Liang Kuo, Sheng-Fu Wang, Yu Liu, Yu-Ting Chiu, Bo-Huan Chen, Chien-Hao Huang, Rong-Nan Chien

**Affiliations:** 1 Department of Gastroenterology and Hepatology, New Taipei Municipal TuCheng Hospital, Tucheng, New Taipei City 236, Taiwan; jeffrey3436@hotmail.com (S.-H.L.); liu870223@gmail.com (Y.L.); ytchiu@cgmh.org.tw (Y.-T.C.);; 2 Division of Hepatology, Department of Gastroenterology and Hepatology, Linkou Chang-Gung Memorial Hospital, Taoyuan 333, Taiwan; 3 College of Medicine, Chang-Gung University, Taoyuan 333, Taiwan

**Keywords:** acute-on-chronic liver failure (ACLF), CLIF-C ACLF, CLIF ACLF-lactate, NACSELD ACLF, specialized liver intensive care unit

## Abstract

Background: Acute-on-chronic-liver failure (ACLF) demonstrates high short-term mortality rates and usually requires intensive care unit (ICU) admission. Accurate prognostication of these patients is pivotal for timely referral for liver transplantation. The superiority of CLIF-C ACLF, CLIF-C ACLF lactate, and NACSELD-ACLF scores in Asian patients with ACLF admitted to an ICU remains inconclusive. Aims: To compare the predictive performance of CLIF-C ACLF, CLIF-C ACLF lactate, and NACSELD-ACLF scores for one-month mortality. Methods: 276 consecutive cirrhotic patients with ACLF admitted to ICU were enrolled. The prognostic values for one-month mortality were assessed by AUROC analysis. Results: The primary cause of cirrhosis in this cohort was alcohol abuse (56.5%). AUROC analysis (95% confidence intervals) demonstrated that CLIF-C ACLF lactate [0.802 (0.747–0.856)] outperformed both CLIF-C ACLF [0.791 (0.733–0.848)] and NACSELD-ACLF [0.673 (0.606–0.740)] in predicting one-month mortality. However, no statistically significant difference was observed between the predictive abilities of CLIF-C ACLF and CLIF-C ACLF lactate. Conclusions: In critically ill cirrhotic patients with ACLF admitted to the hepatology ICU, CLIF ACLF-lactate outperformed CLIF-C ACLF and NACSELD-ACLF in predicting one-month mortality. Nevertheless, no statistically significant difference was observed between CLIF-C ACLF and CLIF-C ACLF lactate. Larger-scale multi-center prospective studies are warranted to validate these results.

## 1. Introduction

Cirrhotic patients face a heightened susceptibility to acute decompensation, which can lead to organ failure and the development of acute-on-chronic liver failure (ACLF) [1,2]. The high short-term mortality rates associated with ACLF often necessitate admission to the intensive care unit (ICU) [3]. Despite critical care and organ support in the ICU, some ACLF patients remain at a heightened risk of rapid clinical deterioration and may require urgent liver transplantation (LT) to improve their chances of survival. Given the narrow transplantation timeframe, having a straightforward prognostic tool at the bedside is invaluable for clinicians, as it assists in making precise decisions regarding patient management and judicious patient prioritization for LT [4,5]. 

Therefore, the EASL-CLIF Consortium developed the CLIF Consortium ACLF score (CLIF-C ACLFs) by simplifying the original CLIF-SOFAs, based on CLIF-C organ failure (OF) scores, which demonstrated a higher prognostic accuracy than the CLIF-SOFA, MELD, MELD-Na, and Child–Turcotte–Pugh (CTP) scores for ACLF patients [6]. It was also externally validated in a single ICU for ACLF patients. Additionally, elevated lactate levels have been linked to unfavorable prognostic outcomes in critically ill patients with ACLF [7,8]. In cases of advanced hepatic and renal conditions, inadequate lactate clearance is often observed. Consequently, the lactate-adjusted CLIF-C ACLF score, known as the CLIF-C ACLF lactate score, was developed based on a multinational study in Germany and Austria [9], which significantly outperformed the original CLIF-C ACLF in predicting 28-day mortality. A multinational study in Portugal and Canada also confirmed its usefulness in predicting ICU mortality in patients with ACLF admitted to ICU [10]. 

On the other hand, the North American Consortium for the Study of End-Stage Liver Disease (NACSELD) initially developed the infection-related ACLF score, defined as the presence of two or more extrahepatic organ failures [11]. These organ failures can be easily assessed at the bedside and include cardiovascular (shock), brain (West Haven Grade 3/4, hepatic encephalopathy), renal (need for dialysis), and respiratory (mechanical ventilation) failures. Subsequent validation in a diverse, multicenter, prospectively enrolled cohort of hospitalized cirrhotic patients, encompassing both infected and uninfected patients, confirmed the score’s ability to predict 30-day survival [12]. This score is designed as a simple bedside tool for predicting 30-day survival in ACLF patients, regardless of infection status [12].

However, limited research has compared the prognostic efficacy of these three well-recognized ACLF scores [13,14,15], and the superior score has not been definitively determined. Considering the substantial short-term mortality rate, as defined in the CANONIC study [1], with a 28-day mortality of at least 33%, our primary objective is to compare the predicting strength of these three scores in terms of one-month mortality among ACLF patient, regardless of their infection status.

## 2. Patient and Method

### 2.1. Diagnosis and Grading of ACLF

a. EASL-CLIF ACLF was diagnosed according to the diagnostic criteria established by the EASL-CLIF consortium. The severity of ACLF was assessed using the criteria outlined by the EASL-CLIF consortium [1,6]. ACLF grade 1 is defined by one of the following criteria: (a) the presence of a single organ failure (e.g., hepatic, coagulation, circulatory, or pulmonary) accompanied by a serum creatinine level of 1.5–1.9 mg/dL and/or West Haven Grade 1 or 2 hepatic encephalopathy; or (b) single renal failure (creatinine level ≥ 2.0 mg/dL) in the absence of other organ failures; or (c) single brain failure with a serum creatinine level of 1.5–1.9 mg/dL. ACLF Grade 2 is characterized by two organ failures, while ACLF Grade 3 is defined by the presence of three or more organ failures.

b. NACSELD-ACLF was defined as the presence of two or more organ failures out of the four categories described [11]. Brain failure was determined based on a West Haven Grade 3 or 4 of encephalopathy. Renal failure was identified as the need for renal replacement therapy, which should be distinguished from acute kidney injury, as recently redefined by the International Ascites Club [16]. Respiratory failure was assessed by the requirement for bilevel-positive airway pressure or mechanical ventilation. Shock was defined as the need for pressor support, a mean arterial pressure <60 mm Hg, or a reduction of >40 mmHg in systolic blood pressure from baseline, despite adequate fluid resuscitation.

### 2.2. Patient Enrollment

**Figure 1:**
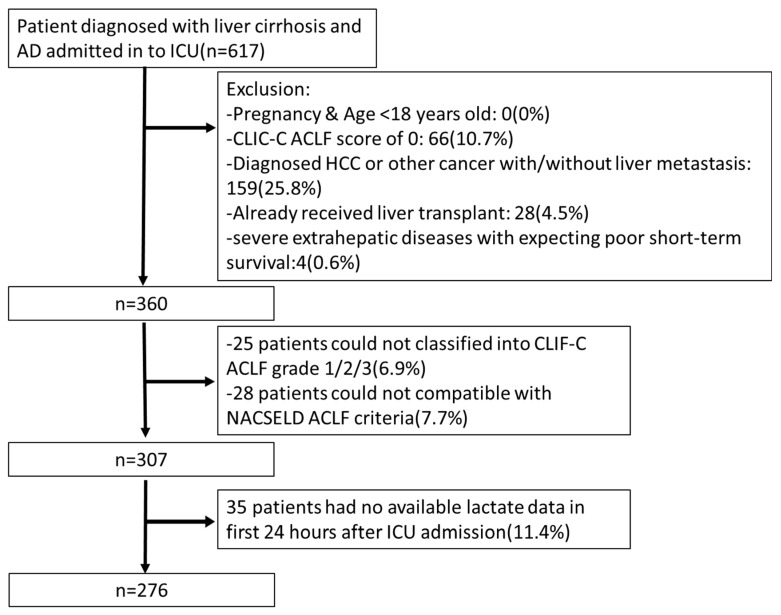
Flow chart demonstrating the process for patient recruitment, inclusion, and exclusion of ACLF cases in the intensive care unit (ICU). AD: acute decompensation.

This study included cirrhotic patients with acute decompensation (AD) who were admitted to the hepato-gastroenterology ICU of Chang-Gung Memorial Hospital, Linkou, Taoyuan, Taiwan, between December 2013 and February 2020, and met the inclusion criteria. The exclusion criteria were as follows (i) under age 18 years old, (ii) pregnancy, (iii) pre-existing hepatocellular carcinoma or other cancers with/without liver metastasis, (iv) prior orthotopic liver transplantation either before or during ICU admission, and (v) severe extrahepatic diseases with an expected poor short-term survival. The study’s design and patient enrollment flow chart are summarized in Figure 1.

### 2.3. Definition of Liver Cirrhosis and Acute Decompensation

Liver cirrhosis was diagnosed based on histopathological confirmation or a composite of compatible clinical features, laboratory tests, and endoscopic findings, as well as radiological imaging [17,18,19,20]. Acute decompensation of cirrhosis is characterized by life-threatening complications [20], including variceal bleeding [21,22], ascites [23], hepatic encephalopathy [24], and bacterial infections such as sepsis [25] or spontaneous bacterial peritonitis [23].

### 2.4. Data Source and Collection

We retrieved and analyzed data from the medical records of patients admitted to the hepato-gastroenterology ICU at Chang-Gung Memorial Hospital, Linkou Medical Center. The collected data included patient demographics, cirrhosis etiology, laboratory test results, vital signs, Glasgow Coma Scale scores, urine output, oxygenation support, mechanical ventilator settings, details regarding the mode of acute decompensation, precipitating events of ACLF at admission, and survival interval. Organ failure and ACLF were classified according to the EASL-CLIF Consortium and North American Consortium criteria, respectively [1,11]. The laboratory data were collected within 24 h of ICU admission and calculated with appropriate formulas.

### 2.5. Primary Outcomes and Follow-Up Period

The primary outcomes were mortalities at one month. Survival of patients after discharge was confirmed through telephone interviews and/or analysis of medical records. We confirmed the survival status of patients after discharge through telephone interviews and/or by analyzing medical records. Each patient was followed until the date of death or until 28 February 2022, whichever occurred first.

### 2.6. Calculation of Prognostic Scores 

The prognostic scores were calculated using their respective formulas proposed in previous studies. The CLIF-C ACLF score = 10 × [0.33 × CLIF-OFs + 0.04 × Age + 0.63 × ln (WBC count) − 2] [6]. The lactate-adjusted CLIF-C ACLF (CLIF-C ACLF lactate score) = CLIF-C ACLFs + 8 × ln (lactate) − 7 [26]. The NACSELD-ACLF = −1.739 − 0.048 × age − 0.555 × WBC + 0.306 × albumin − 0.085 × MELD − 0.402 × infection.

### 2.7. Statistical Analysis

Continuous variables were presented as either the mean ± SD or the median and interquartile range (IQR, 25–75 percentile) depending on their distribution. To compare these variables, we used the independent Student t-test for normally distributed data and the Mann–Whitney U-test for non-normally distributed data. Categorical variables were expressed as frequencies and percentages, and their comparison was performed using the chi-square test. In cases where more than 20% of the cells in the statistical analysis had an expected frequency of less than 5, Fisher’s exact test was employed. The predictive performance of the scores was evaluated by calculating the area under the receiver operating characteristic curve (AUROC) and compared among the CLIF-C ACLF, CLIF-C ACLF lactate, and NACSELD-ACLF scores. A competing risk analysis was not performed due to the limited number of patients who received liver transplantation during the follow-up period. The predictive performance of each score to predict mortality was compared using the Delong test. Statistical analyses were performed using IBM SPSS Statistics 26 (SPSS Inc., Chicago, IL, USA), and a *p*-value < 0.05 was considered statistically significant.

## 3. Result

### 3.1. Patients’ Baseline Characteristics

A total of 276 cirrhotic patients with ACLF were enrolled between December 2013 and February 2020, following the inclusion and exclusion criteria, and subsequently, they were monitored until February 2022 (Figure 1).

Table 1 summarizes the baseline characteristics of the 276 enrolled patients and the comparisons between one-month survivors and non-survivors. First, the one-month mortality rate stood at 36.9% (102/276). The mean age of the cohort was 55.32 ± 13.43 years, with the majority being male. The primary etiology of cirrhosis in the cohort was alcohol abuse, followed by hepatitis B virus infection, hepatitis C virus infection, and other etiologies including non-alcoholic steatohepatitis (NASH) and autoimmune hepatitis. Gastrointestinal bleeding was identified as the predominant cause of ICU admission, maintaining consistency within the survivor subgroup. Conversely, sepsis emerged as the primary reason for ICU admission in the non-survivor group.

In the non-survivor group, notable elevations were evident in several parameters, including the A-a gradient, white cell count, INR, total serum bilirubin, serum blood urea nitrogen, serum creatinine, and serum lactate, as compared to the survivor group. Conversely, the survivor group displayed an elevated serum albumin level when contrasted with the non-survivor group. The utilization of vasopressors, mechanical ventilation, and the presence of underlying end-stage renal disease (ESRD) under hemodialysis did not exhibit statistically significant differences between the survivor and non-survivor groups. Furthermore, the non-survivor group exhibited significantly higher proportions of patients with Grade 3/4 hepatic encephalopathy (HE) when compared to the survivor group. Conversely, the survivor group showed a significantly larger proportion of patients without HE in contrast to the non-survivor group.

### 3.2. Assessment of Predictive Performance: NACSELD ACLF, CLIF-C ACLF, and CLIF-C ACLF Lactate Scores in Predicting the Primary Outcome

**Figure 2:**
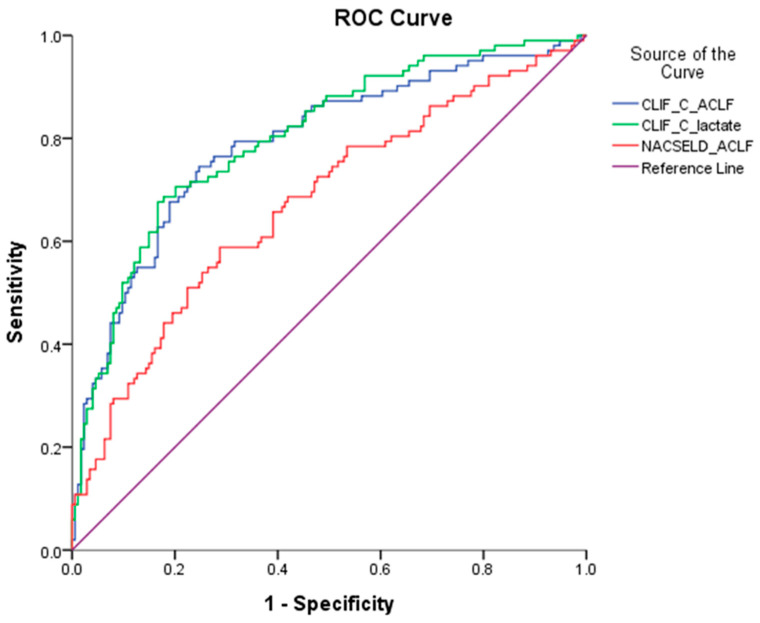
Comparison of the three prognostic scores predicting one-month mortality by AUROC analysis.

The AUROCs of the three prognostic scores (NACSELD ACLF, CLIF-C ACLF lactate, and CLIF-C ACLF scores) were compared concurrently to predict the primary outcomes. As shown in Table 2 and Figure 2, the AUROC (95% confidence intervals) of the three prognostic scores for predicting one-month mortality were as follows: NACSELD ACLF: 0.673 (0.606–0.740), CLIF-C ACLF lactate: 0.802 (0.747–0.856), CLIF-C ACLF: 0.791 (0.733–0.848), respectively. The AUROC analysis revealed that CLIF-C ACLF lactate exhibited the highest predictive strength for one-month mortality, outperforming both CLIF-C ACLF and NACSELD ACLF. However, despite the higher AUROC value for CLIF-C lactate, there was no significant difference in pairwise comparison between CLIF-C ACLF lactate and CLIF-C ACLF.

### 3.3. Cut-Off Values, Sensitivity, and Specificity for the ROC Curve of the Three Prognostic Scores

As presented in Table 3, the cut-off value for CLIF-C ACLF was determined to be 56.79. Above this threshold, the mortality rate was found to be 63.87%, whereas below it, the mortality rate was 16.56%. The diagnostic accuracy of CLIF-C ACLF manifested a sensitivity of 75.29% and a specificity of 74.51%. Similarly, the determined cut-off value for CLIF-C ACLF lactate was identified as 64.04. Above this threshold, the mortality rate was observed to be 70.41%, while below it, the mortality rate was 18.54%. The diagnostic performance of CLIF-C ACLF lactate exhibited a sensitivity of 83.33% and a specificity of 67.65%. Furthermore, the cut-off value for NACSELD ACLF was calculated to be −11.92. Below this value, the mortality rate was 54.55%, and above it, the mortality rate was 25.30%. The diagnostic sensitivity and specificity of NACSELD ACLF in this context were determined to be 71.26% and 58.82%, respectively.

## 4. Discussion

The EASL-CLIF Consortium and the North American Consortium have independently developed predictive models known as the CLIF-C ACLF and NACSELD ACLF scores, respectively [1,11]. CLIF-C ACLF lactate score was developed based on a multinational study conducted in Germany and Austria [10]. However, there has been limited research conducted to compare the prognostic performance of these three influential predictive scores. The determination of the superior predictive score for ACLF patients remains uncertain.

In our study, which enrolled 276 consecutive cirrhotic patients with ACLF admitted to the ICU, the CLIF-C ACLF lactate score demonstrated a superior performance compared to both the CLIF-C ACLF and NACSELD ACLF scores, as indicated by AUROC analysis. However, no statistically significant difference was observed between the CLIF-C ACLF lactate and CLIF-C ACLF scores in pairwise comparisons. We have several hypotheses that may explain our findings. Firstly, the superior predictive performance of the CLIF-C ACLF score over the NACSELD-ACLF score may be attributed to the incorporation of liver and coagulation failure components within the EASL CLIF criteria, which are not included in the NACSELD criteria. Liver and coagulation failure represent the primary organ failures in ACLF and are crucial predictors of mortality. These factors are also considered in the Model for End-Stage Liver Disease (MELD) score [27,28]. Notably, the EASL-CLIF criteria included six organ failures (OFs) whereas NACSELD included only four OFs. Additionally, nearly 30% of patients categorized as EASL-CLIF Grade 3 would not be diagnosed as having ACLF by NACSELD criteria [15]. Supporting this, a study conducted in Brazil also favored the CLIF-C ACLF score, which not only allowed more patients to be diagnosed with ACLF and receive intensive care but also demonstrated a better performance in predicting death at 28 days compared to NACSELD definitions [13].

Secondly, suboptimal lactate clearance can be observed in patients with advanced liver disease and is associated with poor outcomes [7,8,9]. The CLIF-C ACLF lactate score has been shown to be superior to the conventional CLIF-C ACLF score, particularly in predicting short-term mortality in critically ill patients with cirrhosis [9]. Lactate level, which can be easily and rapidly estimated via blood gas analysis at the bedside, serves as a strong indicator of disease severity in critically ill patients with liver cirrhosis [9]. These observations may explain the superior AUROC value observed for CLIF-C ACLF lactate compared to CLIF-C ACLF in cirrhotic patients with ACLF.

However, the results in the current study differ from our previous study conducted in 2021 [26] for several key reasons. First, in the current study, we included a larger number of patients who were enrolled between December 2013 and February 2020, resulting in a longer follow-up period and more accurate 24 h lactate data. This is in contrast to the previous study, which included patients enrolled from November 2012 to April 2015 [26]. It is worth noting that in the previous study, we had mentioned that lactate measurements were more limited [26]. Additionally, the patient population in the current study was predominantly affected by alcohol-related cirrhosis (56.5%), whereas the previous study primarily included patients with HBV-related cirrhosis (40%) [26]. This difference in patient demographics could account for the variation in study outcomes. It is important to note that although the AUROC of CLIF-C ACLF lactate [0.802 (0.747–0.856)] was higher than that of both CLIF-C ACLF [0.791 (0.733–0.848)] and NACSELD-ACLF [0.673 (0.606–0.740)] in predicting one-month mortality, there was no statistically significant difference between the AUROCs of CLIF-C ACLF lactate and CLIF-C ACLF. However, it is worth noting that limited research has compared the prognostic efficacy of these three well-recognized ACLF scores [13,14,15]. Therefore, we acknowledge the necessity for further prospective studies to validate these hypotheses.

We selected one-month mortality as our primary outcome because ACLF patients awaiting transplantation often face the risk of mortality or removal from the transplant list due to rapid clinical deterioration [29,30]. The likelihood of ACLF patients surviving beyond 30 days is notably reduced, especially in cases of increasing organ failure(s) [29,30]. Furthermore, patients who undergo transplantation tend to have a significantly higher one-year survival rate compared to those who do not, particularly when considering ACLF Grade 3 [4,29]. Therefore, the 30-day window emerges as a critical metric for assessing ACLF patients, particularly within the subset experiencing multiple organ failures. Additionally, in the CANONIC study, ACLF is associated with a high short-term mortality rate, defined as the one-month mortality rate, which can range from 23% to 74%, depending on the number of organ failures, despite standard supportive medical treatment [31]. In contrast, acute decompensation without ACLF is associated with a very low one-month mortality rate (<2%).

Precisely identifying the simplest and most reliable prognostic tool for ICU-admitted ACLF patients is of paramount importance in mitigating the high short-term mortality rates within this patient population. This is especially crucial considering that more aggressive treatment modalities, such as liver transplantation, are often considered [4,5]. This matter holds particular relevance in East Asia, where the incidence of HBV-related ACLF has been prevalent [32,33,34]. However, recent successes in deploying potent antiviral agents targeting viral hepatitis have shifted the landscape [33,35]. Presently, alcohol-associated cirrhosis accounts for a substantial portion of the global burden of cirrhosis, both in the United States and worldwide [36,37]. This trend aligns with our study population, where alcoholic-related cirrhosis accounted for 56.5% of cases. The treatment of alcoholic cirrhosis and alcohol-related ACLF is likely to emerge as a crucial healthcare concern in Asia and globally in the future [38,39,40,41]. The development of a precise and simple prognostic model can greatly assist hepatologists and intensivists in accurately predicting prognosis. This, in turn, enables the early identification of high-risk patients who may benefit from liver transplantation [4,5,42,43].

Our study has several limitations. Firstly, being conducted at a single large hepato-gastroenterologic academic center, there exists the possibility of referral bias. Therefore, further collaboration with other ICUs is crucial to enhance the generalizability of our findings. Secondly, it is important to note that the definition of ACLF differs between the EASL-CLIF Consortium and the NACSELD Consortium. The criteria of the NACSELD Consortium exclude patients with acute kidney injury, particularly those in chronic kidney stages 2–3 who are not on dialysis but exhibit poor short-term survival. This disparity in criteria could potentially influence our study results. Thirdly, data collection in our study occurred within 24 h of intensive care unit (ICU) admission, in contrast to the CANONIC study which collected data between Days 3 and 7 to compute the CLIF-C ACLF score. However, we chose to collect data on the first day of ICU admission to ensure consistency. It is worth noting that data obtained at different time points for calculating these prognostic scores may yield different results, and this should be considered in the interpretation of our findings.

## 5. Conclusions

In our study, CLIF ACLF-lactate outperformed CLIF-C ACLF and NACSELD-ACLF in predicting one-month mortality in critically ill cirrhotic patients with ACLF in the hepatology ICU. Nonetheless, there was no significant difference between the CLIF-C ACLF lactate and CLIF-C ACLF scores. These findings suggest that both CLIF-C ACLF and CLIF ACLF-Lactate are valuable tools for predicting outcomes in this patient population. Nonetheless, further prospective studies are warranted to comprehensively compare the prognostic strength of these three predictive models in cirrhotic patients with ACLF who are admitted to ICUs. Such studies will help refine our understanding of the most effective prognostic tools for this high-risk patient group and guide clinical decision-making.

## Figures and Tables

**Table 1:** Demographics of 276 patients with ACLF admitted to ICU.

Patients’ Characteristics	All Patients	Survivors *	Non-Survivors *	*p*-Value
	(276 Patients)	(174 Patients)	(102 Patients)	
Age (mean ± SD years)	55.32 ± 13.43	54.38 ± 13.10	56.91 ± 13.89	0.131
Gender = Male	213 (77.2%)	140 (80.5%)	73 (71.6%)	0.089
Etiology:				
HBV	59 (21.4%)	32 (18.4%)	27 (26.5%)	0.114
HCV	39 (14.1%)	20 (11.5%)	19 (18.6%)	0.101
ALC	156 (56.5%)	108 (62.1%)	48 (47.1%)	0.015
Other †	22 (8.0%)	14 (8.0%)	8 (7.8%)	0.952
Indication of admission:				
Infection	122 (44.2%)	66 (37.9%)	56 (54.9%)	0.006
Bleeding	134 (48.6%)	103 (59.2%)	31 (30.4%)	<0.001
HBV or HCV flare	15 (5.4%)	3 (1.7%)	12 (11.8%)	<0.001
Alcoholism	5 (1.8%)	2 (1.1%)	3 (2.9%)	0.281
Clinical parameters:				
A-a gradient (mmHg)	185.75 ± 150.47	165.92 ± 145.64	219.57 ± 153.24	0.004
White cell count (×1000/μL)	11.66 ± 9.16	10.32 ± 5.30	13.95 ± 13.12	0.009
INR	2.15 ± 1.19	1.75 ± 0.52	2.84 ± 1.62	<0.001
Serum bilirubin (mg/dL)	8.71 ± 10.32	5.27 ± 6.71	14.59 ± 12.55	<0.001
Serum BUN (mg/dL)	40.81± 30.57	34.49 ± 27.68	51.60 ± 32.24	<0.001
Serum creatinine (mg/dL)	1.96 ± 1.57	1.57 ± 1.15	2.64 ± 1.93	<0.001
Albumin (g/dL)	2.6 ± 0.55	2.69 ± 0.53	2.43 ± 0.55	<0.001
Lactate(mmol/L)	4.87 ± 4.52	4.13 ± 3.73	6.15 ± 5.40	0.001
Lactate(mg/dL)	45.06 ± 40.70	38.17 ± 33.84	56.80 ± 48.25	0.001
Use of vasopressors	213 (77.2%)	133 (76.4%)	80 (78.4%)	0.703
Mechanical ventilation use	173 (62.7%)	104 (59.8%)	69 (67.6%)	0.192
Under-hemodialysis	5 (1.8%)	2 (1.1%)	3 (2.9%)	0.281
No HE	96 (34.8%)	81 (46.6%)	15 (14.7%)	<0.001
HE I-II	106 (38.4%)	60 (34.5%)	46 (45.1%)	0.080
HE III-IV	74 (26.8%)	33 (19.0%)	41 (40.2%)	<0.001
Score on admission to ICU mean ± SD:				
MELD	23.94 ± 9.80	19.86 ± 8.01	30.90 ± 8.60	<0.001
CLIF-organ	11.84 ± 2.45	10.82 ± 1.92	13.58 ± 2.28	<0.001
CLIF-C ACLF	55.21 ± 10.90	51.21 ± 9.29	62.03 ± 10.05	<0.001
CLIF-C ACLF-lactate	58.71 ± 13.47	53.58 ± 11.67	67.48 ± 11.73	<0.001
NACSELD-ACLF	10.88 ± 3.38	10.88 ± 3.38	14.13 ± 7.39	<0.001

* Overall survivors vs. non-survivors. † Other etiologies of cirrhosis include NASH (non-alcoholic steatohepatitis) and autoimmune hepatitis, etc. HE: hepatic encephalopathy classified by the West Haven criteria. HBV: chronic hepatitis B virus-related cirrhosis. HCV: chronic hepatitis C virus-related cirrhosis. ALC: alcohol-related cirrhosis. INR: international normalized ratio.

**Table 2:** Comparison of the three prognostic scores to predict one-month mortality by AUROC with pairwise comparison.

			AUROC (95% CI)	Pairwise Sig. Mark		
				A	B	C
NACSELD-ACLF	A	0.673	(0.606–0.740)			
CLIF-C-Lactate	B	0.802	(0.747–0.856)			
CLIF-C-ACLF	C	0.791	(0.733–0.848)			

AUROC: area under receiver operating characteristic curve; The yellow-colored marks reflect a significant difference in pairwise comparison at the significance level (α = 0.05) and are more important than the black ones.

**Table 3:** The cut-off values, sensitivity, and specificity for the ROC curve of the three prognostic scores.

	Cutoff Value	Sensitivity	Specificity
CLIF C ACLF	56.785653945465	0.752873563218	0.745098039216
CLIF C lactate	64.037721683378	0.833333333333	0.676470588235
NACSELD ACLF	−11.92344	0.712643678161	0.588235294118

## Data Availability

Data and study materials will be made available to other researchers upon request via email, along with their IRB approval document.
